# Over 2000-Fold Increased Production of the Leaderless Bacteriocin Garvicin KS by Increasing Gene Dose and Optimization of Culture Conditions

**DOI:** 10.3389/fmicb.2019.00389

**Published:** 2019-03-05

**Authors:** Amar A. Telke, Kirill V. Ovchinnikov, Kiira S. Vuoristo, Geir Mathiesen, Tage Thorstensen, Dzung B. Diep

**Affiliations:** Faculty of Chemistry, Biotechnology and Food Science, Norwegian University of Life Sciences, Ås, Norway

**Keywords:** garvicin KS, leaderless bacteriocins, bacteriocin production, antimicrobial production, lactic acid bacteria, *Lactococcus garviae*, growth media

## Abstract

The leaderless bacteriocin Garvicin KS (GarKS) is a potent antimicrobial, being active against a wide range of important pathogens. GarKS production by the native producer *Lactococcus garvieae* KS1546 is, however, relatively low (80 BU/ml) under standard laboratory growth conditions (batch culture in GM17 at 30°C). To improve the production, we systematically evaluated the impact of different media and media components on bacteriocin production. Based on the outcomes, a new medium formulation was made that increased GarKS production about 60-fold compared to that achieved in GM17. The new medium was composed of pasteurized milk and tryptone (PM-T). GarKS production was increased further 4-fold (i.e., to 20,000 BU/ml) by increasing the gene dose of the bacteriocin gene cluster (*gak*) in the native producer. Finally, a combination of the newly composed medium (PM-T), an increased gene dose and cultivation at a constant pH 6 and a 50–60% dissolved oxygen level in growth medium, gave rise to a GarKS production of 164,000 BU/ml. This high production, which is about 2000-fold higher compared to that initially achieved in GM17, corresponds to a GarKS production of 1.2 g/L. To our knowledge, this is one of the highest bacteriocin production reported hitherto.

## Introduction

The decreasing effectiveness of antibiotics has become a serious worldwide problem due to the emergence of multidrug-resistant bacteria (Bush et al., 2011; Laxminarayan et al., 2016). Despite that, the number of new commercially available antibiotics is dwindling. This is partly due to the fact that developing new antibiotics is a costly process (Holmes and Mauer, 2016), and most biopharma companies are therefore reluctant to invest large money in new antibiotics that soon may be useless because of resistance development. Consequently, there is an urgent need of cost-effective and efficient antimicrobial agents with different killing mechanisms to overcome multidrug-resistant bacteria.

Bacteriocins are ribosomally synthesized antibacterial peptides produced by bacteria, probably as a means to compete for nutrients and habitats (Cotter et al., 2005). So far, hundreds of bacteriocins have been isolated and characterized. Most of them have narrow-spectrum activity, but some are active against a broad-spectrum of bacteria including food-spoiling bacteria as well as important pathogens (Chikindas et al., 1993;de Arauz et al., 2009). Bacteriocins produced by lactic acid bacteria (LAB) are particularly interesting due to LAB’s safe status as they are commonly found in our foods (Grosu-Tudor et al., 2014; Henning et al., 2015) and the gastrointestinal tract of man (Millette et al., 2008) and animals (O’Shea et al., 2009). Most bacteriocins are membrane-active peptides, killing sensitive bacteria by membrane disruption after selective interaction with specific membrane receptors (Oscariz and Pisabarro, 2001; Hasper et al., 2006; Diep et al., 2007; Nissen-Meyer et al., 2009; Tymoszewska et al., 2017). This mode of action is different from most antibiotics, which often act as enzyme-inhibitors (Davis, 1987; Bush and Jacoby, 2010). For this reason, antibiotic-resistant pathogens are often sensitive to bacteriocins, thus making the latter very attractive as alternative or complementary drugs for therapeutic use, especially to fight antibiotic resistance. Nevertheless, poor production is often a bottleneck in large-scaled production of bacteriocins. Previous studies have shown that bacteriocin production can be increased by optimization of growth conditions such as cultivation temperature, pH, aeration and growth medium (Biswas et al., 1991; Parente and Ricciardi, 1994; Aasen et al., 2000; Cabo et al., 2001; Nel et al., 2001; Guerra and Pastrana, 2002; Penna and Moraes, 2002; Tafreshi et al., 2010). In addition, various heterologous expression systems have been reported for increased bacteriocin production (Horn et al., 2004; Kong and Lu, 2014; Jimenez et al., 2015; Jiang et al., 2016; Mesa-Pereira et al., 2017).

Recently, we have reported the identification and characterization of a novel three-peptide bacteriocin called garvicin KS (GarKS), produced by *L. garvieae* KS1546, a strain isolated from raw bovine milk in Kosovo (Ovchinnikov et al., 2016). A gene cluster (*gak*) containing the three structural genes (*gakABC*) and genes likely involved in immunity (*gakIR*) and transport (*gakT*) has been identified in the genome (Ovchinnikov et al., 2016). GarKS is active against a broad spectrum of bacteria such as *Listeria, Staphylococcus, Bacillus, Streptococcus* and *Enterococcus* (Ovchinnikov et al., 2016). Despite its great potential, production of GarKS is relatively moderate under standard laboratory growth conditions. To overcome this problem, we conducted a multi-factorial optimization study that resulted in over 2000-fold increased bacteriocin production. This approach includes medium optimization, increased gene dose and cultivation optimization.

## Materials and Methods

### Bacterial Strains and Growth Conditions

All bacterial strains and plasmids used in this study are listed in Table 1. Unless otherwise stated, the native bacteriocin producer *L. garvieae* KS1546 was grown in M17 broth supplemented with 0.5% glucose (GM17) under static condition at 30°C. NEB^®^ 10-beta *E. coli* (New England Biolabs, Beverly, MA, United States) was grown in Luria-Bertani (LB) broth with shaking (200 rpm) at 37°C. Bacterial culture media and supplements were obtained from Oxoid Ltd. (Hampshire, United Kingdom). When necessary, erythromycin (Sigma-Aldrich Inc., St. Louis, MO, United States) was added at 200 μg/ml for *E. coli* and at 5 μg/ml for LAB strains.

**Table 1:** Bacterial strains, plasmids and primers used in this study.

Strain, plasmid or Primer	Description	Source/reference
*Strains*		
*L. garvieae* KS1546	Wild type strain, native GarKS bacteriocin producer	[31]
*L. garvieae* KS1546-pA2T	*L. garvieae* KS1546 containing the recombinant plasmid pA2T	This study
*L. lactis* IL 1403-pA2T	*L. lactis* 1403 containing the recombinant plasmid pA2T	This study
*L. garvieae* KS1546-pMG	*L. garvieae* KS1546 containing the empty plasmid pMG36e	This study
*L. lactis* IL 1403-pMG	*L. lactis* IL 1403 containing the empty plasmid pMG36e	This study
*Escherichia coli* NEB 10-beta	Subcloning host strain	New England Biolab
*Plasmids*		
pMG36e	Em^R^, *E. coli*-*Lactococcus* shuttle vector	[48]
pABC	pMG36e containing the structural genes *gakABC*, Em^R^	This study
pA2T	pMG36e containing the entire *gak* cluster; Em^R^	This study
*Primers*		
gakF	5′-CGTAATTCGAGCTCCACCTC TGCTGTTTTTC-3′	This study
gakR	5′-AGACTTTGCAAGCTTGCAAT ATTACGTTTGTGGG-3′	This study
gakR1	5′-AGACTTTGCAAGCTTTTAATCC TGACTCATCAGATATTC-3′	This study
gakSeqF	5′-GTACATAGTACCTCAAAATTAT TTGAGC-3′	This study
gakseqF1	5′-GCAGAGCTTTAGTGTGGGAT-3′	This study
gakseqF2	5′-CGCTATTGCTTCTGAATATATA GTGGAC-3′	This study
gakseqF3	5′-GGCACTTTTACAAGAAATAGG ACT-3′	This study
gakseqR	5′-AGTAATTGCTTTATCAACTGCT GC-3′	This study
pMGF	5′-CATCCTCTTCGTCTTGGTAGC-3′	This study
pMGR	5′-GGCAGCTGATCTCAACAATG-3′	This study


### Growth Media for GarKS Production

The influence of different growth media on GarKS production was assessed in batch cultures under static condition at 30°C. Following commercial complex media were used: GM17, deMan, Rogosa and Sharpe (MRS), Todd-Hewitt (TH) and Brain Heart Infusion (BHI). To make new milk-based medium formulations, skim milk (5%, w/v) or pasteurized skim milk was combined with an equal volume of GM17, MRS, TH, and BHI, or with tryptone (10% w/v). Skim milk (SM) was prepared by using milk powder (Oxoid, United Kingdom) while pasteurized milk (PM) was obtained from a dairy company in Norway, Q-milk.

### DNA Manipulation

The *gak* cluster responsible for production of GarKS was amplified from genomic DNA of *L. garvieae* KS1546 using Phusion High-fidelity DNA polymerase (New England Biolabs, United Kingdom) and the primers gakF and gakR1 (Table 1). The genes *gakABC* encoding the three peptides constituting GarKS were amplified using the primers gakF and gakR (Table 1). Restriction sites SacI and HindIII were introduced at the 5′end of forward and reverse primers. NEBuilder HiFi DNA assembly cloning kit (New England Biolabs) was used to assemble the PCR fragments into the plasmid pMG36e (van de Guchte et al., 1989). Plasmid DNA was amplified in *E. coli* NEB^®^ 10-beta before being transferred into *L. garvieae* KS1546 or *L. lactis* IL1403 cells using a Gene Pulser^TM^ (Bio-Rad Laboratories, Hercules, CA, United States). Primers used in this study were obtained from Life Technologies AS (Thermofisher Scientific, Oslo, Norway). The integrity of all recombinant plasmids was confirmed by Sanger DNA sequencing (GATC Biotech AG; Constance, Germany), which were sequenced using primers gakseqF, gakseqF1, gakseqF2, gakseqF3, gakseqR, pMGF, and pMGR (Table 1).

### Optimization of Bacteriocin Production in Bioreactor Conditions

The effects of pH and aeration on GarKS production were tested at various constant pHs (5, 6, and 7), and at controlled aeration in a fully automated 2.5 L Minifors 1 bioreactor (Infors AG, Switzerland). The pH was controlled by automatic addition of 5 M HCl or 5 M NaOH. The aeration was maintained by purging sterile air into culture medium. Temperature (30°C) and agitation speed of 150 rpm were maintained constant for all experiments. Samples of 2 ml were withdrawn aseptically every 2 h for determination of bacteriocin production and cell growth (see below).

### Determination of Bacteriocin Production and Cell Growth

Bacteriocin activity was measured from heat-inactivated (100°C for 10 min) cell-free culture supernatants. Bacteriocin activity was quantified using a microtiter plate assay as previously described (Jimenez et al., 2015; Ovchinnikov et al., 2016). One bacteriocin unit (BU) was defined as the minimum amount of the bacteriocin that inhibited at least 50% of growth of the indicator (*L. lactis* IL103) in a 200 μl culture volume. Growth curve was determined by measuring turbidity of culture at OD_600_ every 30 min for 24 h or by counting colony forming units (CFU) from serially diluted bacterial cultures on agar plates. Synthetic GarKS peptides were purchased from Pepmic Co., LTD., China, with GarA of about 85% purity and GarB and GarC of at least 95% purity. (Higher purity of GarA could not be synthesized due to constant problems occuring during synthesis/purification). GarKS composed of these synthetic peptides in equal amounts (1:1:1, w/v), has a specific activity of 130–140 BU/μg. This specific activity was used to estimate the amount of GarKS produced in cultures.

## Results

### GarKS Production in Complex Media

**FIGURE 1:**
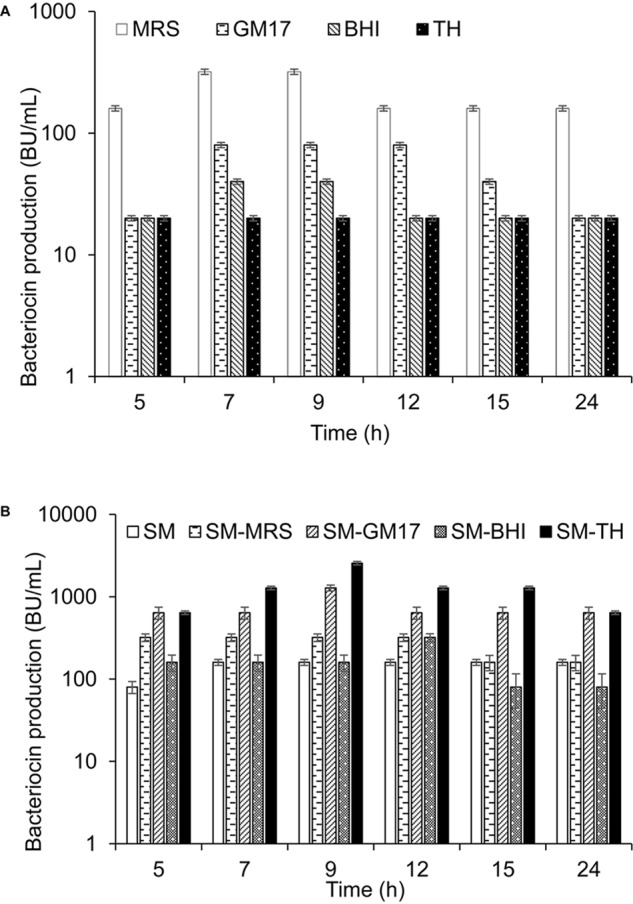
GarKS production by the native producer in different complex growth media **(A)**, and in skim milk (SM) combined with complex growth media **(B)**. Each culture was started by adding 1% (v/v) culture inoculum to 5 ml growth medium and then incubated at 30°C without shaking. Bacteriocin activity was measured at different time points. Standard deviations were based on triplicate assays.

Two different ways were used to describe the production of garvicin KS in a culture: production per ml expressed as bacteriocin unit per ml (BU/ml), and production per 10^8^ cells expressed as BU/10^8^ cells, the latter being referred to as specific production. *L. garvieae* KS1546 (hereafter shortened as KS1546) was routinely grown in the complex medium GM17 at 30°C without agitation, and GarKS production was typically of 80 BU/mL after 7–12 h growth. To examine whether the level of production was medium-dependent, KS1546 was grown in different complex media (MRS, BHI, and TH). Highest production was found between 7–12 h of growth in all tested media except for TH where bacteriocin production appeared constantly low for all time-points tested (Figure 1A). Relative to GM17, GarKS production increased 2 to 4-fold in MRS, while it was about 2 to 4-fold less in BHI and TH (Figure 1A). Cell growth was best in GM17 (30 × 10^8^ cells/ml) but poorest in MRS (10 × 10^8^ cells/ml) after 24 h at 30°C (Table 2). The growth in MRS gave highest specific production, 32 BU/10^8^ cells, which is about 12 fold higher than that obtained when cells were grown in GM17 (2.7 BU/10^8^ cells).

**Table 2:** Influence of growth media, increased gene dose and culture conditions on bacteriocin production.

Strain	Growth medium	Bacteriocin production (BU/ml)^c^	Cell growth (×10^8^ cells/ml)^c^	Specific activity (BU/10^8^ cells)^d^
Native producer *L. garvieae* KS1546	GM17^a^	80 (1)	30 (1)	2.7 (1)
	MRS^a^	320 (4)	10 (0.3)	32 (12)
	BHI^a^	20 (0.25)	15 (0.5)	1.3 (0.5)
	TH^a^	20 (0.25)	20 (0.7)	1.0 (0.4)
	SM^b^ (10%, w/v)	160 (2)	2 (0.1)	80 (30)
	Tryptone^a^ (10%, w/v)	80 (1)	3 (0.1)	27 (10)
	SM-TH^b^	2600 (32.5)	29 (1)	90 (33)
	SM-GM17^b^	1280 (16)	30 (1)	43 (16)
	SM-MRS^b^	320 (4)	28 (0.9)	11 (4.2)
	SM-BHI^b^	160 (2)	29 (1)	5.5 (2)
	SM-T^b^	2600 (32.5)	30 (1)	87 (32)
	SM-T-YE^b^	1300 (16)	30 (1)	43 (16)
	PM-T^b^	5100 (64)	35 (1.2)	146 (54)
The recombinant producer *L. garvieae* KS1546-pA2T	PM-T^b^ (uncontrolled pH)	20,000 (259)	35 (1.2)	570 (210)
	PM-T^b^ (constant pH 5)	2600 (32,5)	32 1.1)	81 (30)
	PM-T^b^ (constant pH 6)	82,000 (1025)	70 (2.3)	1170 (430)
	PM-T^b^ (constant pH 7)	41,000 (512)	65 (2.2)	630 (230)
	PM-T^b^ (constant pH 6 and aeration)	164,000 (2050)	100 (3.3)	1640 (610)


*The bacteriocin activity and cell growth from complex growth media (a) and milk based media (b) were measured after 7 and 9 h of incubation, respectively. ^*c*^: Values are average of triplicate assays, numbers in parentheses are fold increased or decreased in relation to the value in GM17. ^*d*^: numbers in parentheses are fold increased or decreased in relation to the value in GM17*.

### GarKS Production Increased in Milk-Based Media

It is well known that bacteria are ecologically adapted to the environments where they normally thrive. Since the producer KS1546 was isolated from raw milk (Ovchinnikov et al., 2016), we examine the possibility to use skim milk (SM) as growth medium. Bacteriocin production was increased 2-fold in SM (160 BU/ml) compared to GM17 (Figure 1B). However, cell growth was remarkably poor in skim milk (2 × 10^8^ cells/ml) (Table 2), resulting in a relatively high specific production, 80 BU/10^8^ cells. The poor growth suggests that some growth factors were present in complex media but absent in SM. Therefore, we tested the mixtures (50:50; v/v) of skim milk and complex media (GM17, MRS, BHI, and TH). As a result, the bacteriocin production was increased 16 times in skim milk combined with TH (SM-TH) and 8 times in SM-GM17, compared to the production in skim milk (SM) (Table 1 and Figure 1B). The bacteriocin production in SM-TH and SM-GM17 was 2600 BU/ml and 1280 BU/ml after 9 h of incubation, respectively. On the other hand, no significant increase of GarKS in SM-MRS (320 BU/ml) and SM-BHI (160 BU/ml) was found in all time points (Figure 1B). All medium formulations gave similar cell densities, i.e., between 28 × 10^8^-30 × 10^8^ cells/ml (Table 1). In terms of specific production, SM-TH gave the highest while SM-BHI gave the lowest, 90 BU/10^8^ cells and 5.5 BU/10^8^ cells, respectively.

**FIGURE 2:**
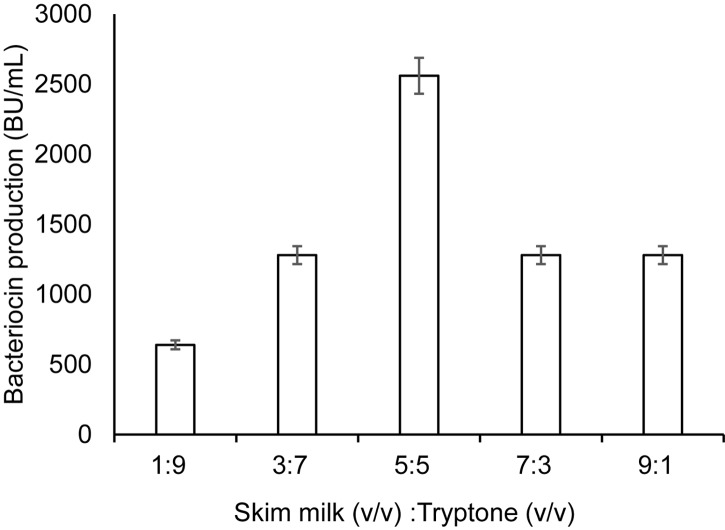
Bacteriocin production in a medium composed of skim milk and tryptone. Different ratios of skim milk and tryptone were made in the formulation by mixing an increasing portion of skim milk (10%; w/v; from 1 volume to 9 volumes) with a corresponding decreasing portion of tryptone (10%, w/v; 9 volumes to 1 volume). For growth conditions, see legend in Figure 1. The bacteriocin activity was measured after 9 h of culture incubation. Standard deviations were based on triplicate assays.

The results above indicate that bacteriocin production was significantly influenced by some specific factor(s)/nutrient(s), which are present in TH and GM17, but absent in MRS and BHI. Tryptone, a tryptic digest of milk protein casein (Oh et al., 1995), is one of the nutrients found in GM17 and TH, but not in MRS and BHI. The final concentration of tryptone in GM17 and TH broth is 0.5 and 2%, respectively. To examine whether tryptone could improve bacteriocin production in combination with SM, we made formulations with different v/v ratios of SM and 10% tryptone (w/v). Highest bacteriocin production (about 2,600 BU/ml) was achieved when they were mixed in equal volumes (50%; v/v); this mixture had a final concentration of tryptone at 5% (w/v) (Figure 2). Under these circumstances, final cell density was comparable to that in GM17, i.e., about 30 × 10^8^ cells/ml (Table 2), giving a relatively high specific production, 87 BU/10^8^ cells, which is comparable to that in SM-TH (90 BU/10^8^ cells). The formulation composed of SM (50%; v/v) and a final 5% of tryptone (w/v) is hereafter called SM-T.

Yeast extract is a rich source of vitamins, minerals, and amino acids, which often improves bacterial growth. We examined the effect of yeast extract (YE) in combination with SM-T. The resulting formulation, SM-T-YE (SM-T containing 1% (w/v) yeast extract) yielded the same cell density as in SM-T (30 × 10^8^ cells/ml), but bacteriocin production was reduced by 50% (Table 2). Yeast extract was therefore excluded from the growth medium.

Although SM-T appeared as a good medium for the producer, we constantly encountered the problem associated with caramelization of milk sugars in skim milk during autoclaving, which might have detrimental effects on nutrition value. To avoid this problem, the autoclaved skim milk in SM-T was replaced with an equal amount of pasteurized skim milk, resulting in a new medium termed pasteurized milk–tryptone (PM-T). The content in pasteurized milk (Q-milk) according to the manufacturer (Q-Meieriene AS, Bergen, Norway) is, g/l: fat, 5; carbohydrate, 45; protein, 35; salt, 1; calcium, 1.3; vitamin B_2_, 0.001; and vitamin B_12_, 0.7 × 10^-5^. Indeed, cell growth in PM-T was slightly increased from 30 × 10^8^ cells/ml to 35 × 10^8^ cells/ml, and GarKS production was increased two-fold in comparison to that in SM-T (Table 2).

Taken together, compared to other media analyzed so far, PM-T gave the best results in all aspects assessed: highest production (5100 BU/ml), highest cell density (35 × 10^8^ cells/ml) and highest specific production (146 BU/10^8^ cells).

### GarKS Production Increased by Higher Gene Dose

The three structural genes (*gakABC*) encoding the three peptides that constitute GarKS are clustered with genes probably involved in immunity (*gakIR*) and transport (*gakT*). First we explored the possibility to increase bacteriocin production by increasing only the gene dose of structural genes *gakABC* in the native producer. The recombinant plasmid pABC carrying structural genes *gakABC* was constructed to deliver high gene dose in the native producer (Table 1). However, we failed to get any transformants even after several attempts. Similar negative result (i.e., no transformants) was obtained when we attempted to transfer pABC into the heterologous host *L. lactis* IL1403 (data not shown). Probably, increased gene dose of the structural genes might override the immunity or/and the transporter in the native producer, leading to toxicity to cells. Consequently, the plasmid pA2T carrying the entire *gak* locus including the genes involved in immunity and transport was constructed pA2T (Table 1). The resulting plasmid was first transferred into *L. lactis* IL1403 to assess the functionality of the locus. As expected, transformation was successful and bacteriocin production was detected in transformed cells (data not shown), confirming the functionality of the *gak* locus. Next, the plasmid was transferred into the native KS1546 and the clone (KS1546-PA2T) was assessed for bacteriocin production. Using PM-T as growth medium, GarKS production by the recombinant producer KS1546-pA2T was found to increase to 20,000 BU/mL, which is about 4 times more than the production without increased gene dose (native KS1546 in PM-T), and about 250-fold more than that initially obtained in GM17 (native KS1546 in GM17) (Table 2).

**FIGURE 3:**
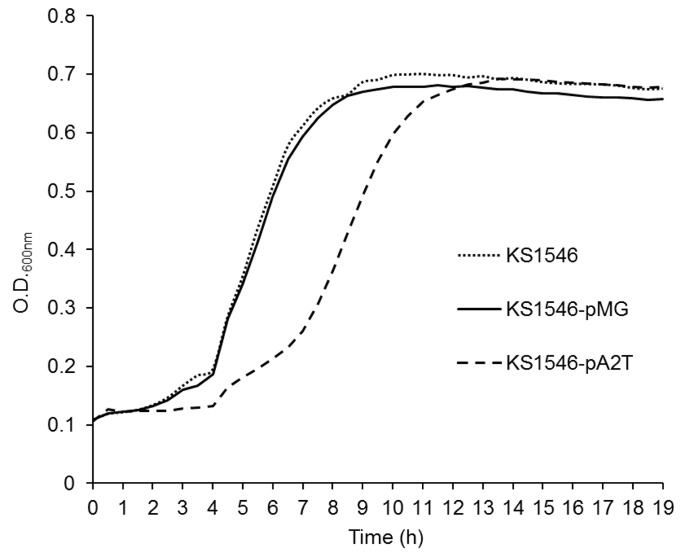
Temporal growth profile of the recombinant producer (KS1546-pA2T), and the native producer with empty plasmid (KS1546-pMG) or without plasmid (*L. garvieae* KS1546). Data were acquired from triplicate assays. Standard deviations are within a range ± 0.01 to ± 0.05.

To compare their growth profiles, the native and recombinant producers were grown in MRS under similar growth conditions and their growth was measured spectrophotometerically. (The medium PM-T was not used due to the turbidity of milk particles causing problem for spectrophotometric reading). The recombinant producer KS1546-pA2T showed a prolonged lag growth phase compared to the native GarKS producer or the native GarKS producer containing the empty plasmid. Nevertheless, KS1546-pA2T reached eventually about the same high cell density as the wild type control cells when it entered stationary growth phase (see Figure 3).

The plasmid map of pABC **(A)** and pA2T **(B)**, which were used to increase the gene dose of the structural genes (*gakABC*) and the *gak* cluster in the native producer, respectively.

### Optimization of Culture Conditions in a Bioreactor Increased GarKS Production

**FIGURE 4:**
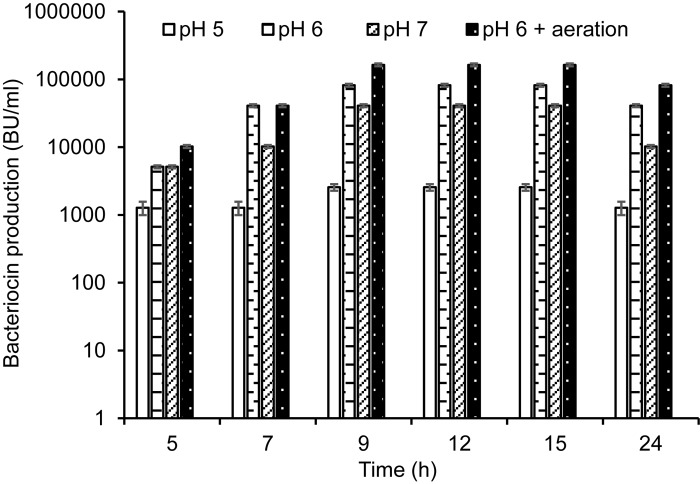
GarKS production of the recombinant producer (KS1546-pA2T) in cultivation at constant pH (pH at 5, 6, or 7) or at constant pH 6 and aeration (50-60% dissolved oxygen). Each culture was started by adding 2% (v/v) culture inoculum in 1.5 l of PM-T medium containing erythromycin at final concentration of 5 μg/ml. Standard deviations were based on triplicate assays.

The initial pH at 7 was declined to 4.8 when the recombinant producer KS1546-pA2T was grown in PM-T for 6–7 h at 30°C (data not shown). To examine whether pH reduction could have a negative impact on cell growth and bacteriocin production, we grew the recombinant producer (KS1546-pA2T) in PM-T in a bioreactor with constant pH at 5, 6, or 7. Indeed, pH had a great impact on cell growth and bacteriocin production. Highest cell growth (70 × 10^8^ cells/ml) and bacteriocin production (82,000 BU/ml) were found at constant pH 6 (Table 2). Bacteriocin production measured at all time-points was also highest at constant pH 6 (Figure 4). Cell growth and bacteriocin production were lowest at constant pH 5. The impact of constant pH also reflects in specific production, that amounted to 1170 BU/10^8^ cells at pH 6 but 81 BU/10^8^ cells at pH 5.

Aeration is defined as dissolved oxygen (DO) percentage in a culture medium. We observed that the initial DO level at 50–60% was declined to 10% after 2 hours of cell growth in PM-T medium and at constant pH 6. The effect of aeration on GarKS production was therefore examined by purging the atmospheric sterile air into the growth medium. With aeration kept at 50–60% and constant pH at 6, highest cell growth (100 × 10^8^ cells/ml) as well as highest bacteriocin production (164,000 BU/ml) and highest specific production (1640 BU/10^8^ cells) were obtained (Table 2 and Figure 4). This level of bacteriocin production (164,000 BU/ml) was about 2000-fold more than the initial production in GM17 which was 80 BU/ml, and about 600-fold more in terms of specific production (1649 BU/10^8^ cells vs. 2.7 BU/10^8^ cells, respectively).

We have previously shown that synthetic GarKS is functionally comparable to the biologically produced counterpart (Ovchinnikov et al., 2016). Synthetic GarKS has a specific activity of 130–140 BU/μg. Hence, the production of 164,000 BU/ml is equivalent to 1.2 g GarKS per liter which is a level of commercial importance.

## Discussion

GarKS is potent against a set of important pathogens including *Staphylococcus, Bacillus, Listeria, Streptococcus*, and *Enterococcus*, making it very attractive in diverse antimicrobial applications from food to medicine. Unfortunately, as also for many other bacteriocins, GarKS is produced at relatively low levels under normal laboratory growth conditions (Ovchinnikov et al., 2016). The low production by the native producer can dramatically hamper potential applications of GarKS as industrial use of bacteriocins requires high and cost-effective production. We have shown in this study that optimization of bacteriocin production by a bacterial strain is a multi-factorial process, which involves a systematic evaluation of nutritional ingredients and growth conditions e.g., temperature, pH, and aeration. The type of growth medium is probably one of the key factors in bacteriocin production (Guerra et al., 2005). The complex media e.g., GM17, MRS, BHI, and TH have been used in cultivation of LAB because they give relatively good cell growth under laboratory conditions but not necessary for bacteriocin production (Mataragas et al., 2004). This was also illustrated in our study: GarKS production was best in MRS (320 BU/ml) but poorest in BHI and TH (both 20 BU/ml) while the cell growth appeared about in the same range in these media (10-20 × 10^8^ cells/ml).

To choose the optimal medium for bacteriocin production is often an empirical matter. The components from complex media influencing bacteriocin production are often elusive and the outcomes might vary significantly dependent on the type of producers. Nevertheless, some media components have been shown to enhance bacteriocin production by inducing stress conditions due to nutrient limitation (Verluyten et al., 2004) or stabilizing the bacteriocin molecules (Herranz et al., 2001). The use of commercial complex media (e.g., MRS) is not a cost-effective approach for large-scale bacteriocin production. For instance, culture medium could account for up to 30% of the total production cost in commercial biomolecule production (Rivas et al., 2004). Accordingly, high costs of complex media will reduce attractiveness of bacteriocins for commercial application. Our bacteriocin producer is a strain of *L. garvieae* isolated from raw milk and it has the capacity to ferment milk-associated sugars such as lactose and galactose while another strain of *L. garvieae* isolated from intestine of Mallard duck can not (Ovchinnikov et al., 2016). Milk is a low-cost product relative to complex media and could be an ideal medium for GarKS producer. However, the native producer appeared to grow poorly in sole skim milk. Skim milk is enriched in lactose and galactose as carbon source but does not contain easily accessed nitrogen-containing components for bacteria. Thus, the combination of tryptone and pasteurized skim milk, which was found best for cell growth, was in line with the notion that tryptone serves as an enriched source of nitrogen. Further, this formula also increased bacteriocin production over 30 fold compared to the growth in GM17.

Increase of gene dose is another means to enhance the production of biomolecules (Nijland et al., 2010). In the present study, we observed a 4-fold increase in bacteriocin production when a plasmid carrying the entire *gak* locus was introduced into the native producer. Interestingly, when we attempted to increase gene dose by introducing the structural genes only (using the plasmid pABC), no transformed cells were obtained. One possible explanation for this negative outcome is that expression of genetic determinants involved in bacteriocin biosynthesis is often highly fine-tuned to secure immunity and efficient export. The extra gene dose of the structural genes alone might override either immunity and/or transporter proteins, leading to toxicity and cell death. It is worth mentioning that most bacteriocins are expressed with a leader sequence which is necessary not only for export but also to keep the bacteriocins in an inactive form before export. For leaderless bacteriocins, such as GarKS, they are produced in mature active forms before export, therefore an intracellular dedicated protection mechanism (immunity) is crucial for cell survival.

We and others have observed that bacteriocin production by a certain strain is unstable, and dependent on the culture conditions applied (Diep et al., 2000; Criado et al., 2006). Consequently, different growth parameters were examined to optimize the production of GarKS. LAB are well known for reducing culture pH due to lactic acid production (Bartkiene et al., 2015) and this is also true for the GarKS producer. We found that culture conditions with constant pH 6 favors the cell growth and a high level of GarKS production. Similarly, optimal nisin production has been reported at constant pH 6.5 (Gonzalez-Toledo et al., 2010). The availability of oxygen also has a great influence on microbial cell growth and metabolic activities (Garcia-Ochoa and Gomez, 2009). Microorganisms vary with respect to their requirements and tolerance toward molecular oxygen. *L. garvieae* is a facultative anaerobic microorganism and its metabolic activities have been reported to differ between aerobic and anaerobic conditions (Delpech et al., 2017). We observed that the controlled aeration had a positive effect on the cell growth and bacteriocin production. Similar results have also been observed for other bacteriocins. For example, nisin A production by *L. lactis* UL719 was enhanced with aeration (Amiali et al., 1998). On the other hand, aeration has also been reported to be antagonistic to the production of lactosin S (Mortvedt-Abildgaard et al., 1995) and LIQ-4 bacteriocin (Kuhnen et al., 1985), suggesting that the effect of aeration on bacteriocin production is strain-dependent.

In terms of cost-effectiveness, the medium PM-T contained tryptone which is a relatively costly component; therefore we are searching for alternatives to replace tryptone. In preliminary studies, we have tested a chicken hydrolysate (processed from a waste product from meat industry) as an alternative low-cost protein source to produce GarKS. We found that the recombinant producer grew well in a medium based on Pasteurized milk and chicken hydrolysate (PM-CH), yielding a cell density of 30 × 10^8^ cells/ml. However, although GarKS production in PM-CH was 8 times better than in the complex media GM17, the production was 8 times less than in PM-T. Thus, further studies are necessary to optimize a PM-CH-based medium in order to achieve high and cost-effective bacteriocin production.

Low bacteriocin production is often a bottle-neck in large-scaled production of bacteriocins for commercial use. Optimization of bacteriocin production is therefore an important research field to better exploit the antimicrobial potential of bacteriocins, especially with regard to the decreasing effects of antibiotics in infection treatments due to the global emergence of antibiotic resistance. In the present study we have achieved a very high level of GarKS production, amounting to 164,000 BU/ml, by combining medium optimization, increased gene dose and culture condition optimization. This amount is about 2,000 times higher compared to the initial production in GM17 (80 BU/ml). A production of 164,000 BU/ml is equivalent to 1.2 g GarKS per liter. This estimation was based the activity of synthetic GarKS peptides which have an activity comparable to the biological ones (Ovchinnikov et al., 2016). Ideally, the activity should be purified and quantified directly by chromatography or immuno-detection approach. However, purification was a challenging task due to their relatively small sizes (32-34 aa), an inherent high hydrophobicity (especially GarA), the multi-peptide property, and not the least, the presence of milk particles in growth medium (PM-T). In fact, purification of GarKS in a previous work was heavily assisted by genetic data to help identity the peptides of GarKS (Ovchinnikov et al., 2016). Immuno-detection was also difficult due to their small sizes and lack of antigenic property (i.e., too hydrophobic). However, given that this estimation is correct, the production of 1.2 g GarKS per liter is, to our knowledge, one of the highest bacteriocin production achieved so far. In comparison, nisin production has been reported to 0.40–0.80 g/L by *L. lactis* grown in a medium composed of equal volume of skim milk and complex media GM17 (de Arauz et al., 2009). Finally, our study and others’ have shown that optimization of bacteriocin production is an empirical and multi-factorial process and that it is highly strain-dependent. Only by systematic evaluation of different aspects influencing growth and gene regulation one can find conditions suitable for high levels of production.

## Author Contributions

AT and KO were involved in experimental design, conducted experiments, collected data and drafted the manuscript. KV was involved in fermentation in bioreactors, GM in cloning, TT in project guidance, DD in experimental design, project leadership and paper writing.

## Conflict of Interest Statement

The authors declare that the research was conducted in the absence of any commercial or financial relationships that could be construed as a potential conflict of interest.
